# Polarization Control in Integrated Graphene-Silicon Quantum Photonics Waveguides

**DOI:** 10.3390/ma15248739

**Published:** 2022-12-07

**Authors:** Simone Cammarata, Andrea Fontana, Ali Emre Kaplan, Samuele Cornia, Thu Ha Dao, Cosimo Lacava, Valeria Demontis, Simone Iadanza, Valerio Vitali, Fabio De Matteis, Elena Pedreschi, Guido Magazzù, Alessandra Toncelli, Franco Spinella, Sergio Saponara, Roberto Gunnella, Francesco Rossella, Andrea Salamon, Vittorio Bellani

**Affiliations:** 1 Istituto Nazionale di Fisica Nucleare (INFN) Sezione di Pisa, 56127 Pisa, Italy; 2 Dipartimento di Ingegneria dell’Informazione, Università di Pisa, 56122 Pisa, Italy; 3 Istituto Nazionale di Fisica Nucleare (INFN) Sezione di Pavia, 27100 Pavia, Italy; 4 Dipartimento di Ingegneria Industriale e dell’Informazione, Università di Pavia, 27100 Pavia, Italy; 5 Dipartimento di Scienze Fisiche, Informatiche e Matematiche, Università di Modena e Reggio Emilia, 41125 Modena, Italy; 6 Istituto Nazionale di Fisica Nucleare (INFN) Sezione di Roma Tor Vergata, 00133 Roma, Italy; 7 Dipartimento di Ingegneria Industriale, Università di Roma Tor Vergata, 00133 Roma, Italy; 8 NEST, Scuola Normale Superiore, Istituto Nanoscienze-CNR, 56127 Pisa, Italy; 9 Centre for Advanced Photonics and Process Analysis, Munster Technological University, Tyndall National Institute, T12 P928 Cork, Ireland; 10 Optoelectronics Research Center (ORC), University of Southampton, Southampton SO17 1BJ, UK; 11 Dipartimento di Fisica, Università di Pisa, 56127 Pisa, Italy; 12 Istituto Nazionale di Fisica Nucleare (INFN) Sezione di Perugia, 06123 Perugia, Italy; 13 Scuola di Scienze e Tecnologie, Università di Camerino, 62032 Camerino, Italy; 14 Dipartimento di Fisica, Università di Pavia, 27100 Pavia, Italy

**Keywords:** quantum photonics, silicon-graphene heterostructure, polarization control

## Abstract

We numerically investigated the use of graphene nanoribbons placed on top of silicon-on-insulator (SOI) strip waveguides for light polarization control in silicon photonic-integrated waveguides. We found that two factors mainly affected the polarization control: the graphene chemical potential and the geometrical parameters of the waveguide, such as the waveguide and nanoribbon widths and distance. We show that the graphene chemical potential influences both TE and TM polarizations almost in the same way, while the waveguide width tapering enables both TE-pass and TM-pass polarizing functionalities. Overall, by increasing the oxide spacer thickness between the silicon waveguide and the top graphene layer, the device insertion losses can be reduced, while preserving a high polarization extinction ratio.

## 1. Introduction

Quantum Key Distribution (QKD) enables the provably secure distribution of private keys, relying on quantum mechanics fundamental principles [1]. Silicon photonics polarization-encoded QKD systems working in the telecom C-band present significant advantages compared to bulky systems in terms of cost, scalability, power consumption and reliability [2,3]. The integrated QKD chip should implement an integrated, linearly polarized, single-photon source, followed by an electrically controlled polarization rotator that aligns the photon polarization along one of the four axes (0, 90, −45, 45) of two non-orthogonal polarization bases [3]. These photons are then transmitted via a polarization-maintaining grating coupler over an optical fiber to the QKD chip [4]. Integrated silicon photonics has been used to implement and test several quantum optical circuits, including large multidimensional quantum entanglement circuits and transmitters for polarization-encoded QKD [5].

Graphene, 2D nanomaterials and semiconductor nanowires can be used to build nanodevices that are able to coherently and dynamically manipulate the photon polarization [6]. In 2D material-based nanodevices, the conductivity regime can be precisely set by exploiting the electrostatic doping via gate control, switching between regimes in which inter-band chiral transitions, rather than surface plasmon polariton resonances, dominate the polarization response [7,8]. Moreover, 2D quantum nanomaterials [9], such as graphene and other 2D materials [10], are expected to play a key role in the development of next-generation quantum photonics platforms. Graphene can selectively support either TE and TM polarization modes, depending on its Fermi level and photon energy, with the additional advantage of broadband operation because of its gapless optical spectrum [11]. By placing it directly on top of an optical fiber, a TE polarizer working from visible to NIR with a large extinction ratio (~27 dB at 1.55 μm) has been demonstrated [12]. Silicon-based photonic-integrated circuits (PICs) are becoming important in a broad range of applications, ranging from datacom to quantum technologies [13]. However, this platform still presents some limitations, since the silicon-on-insulator (SOI) waveguides suffer from large polarization birefringence due to the high refractive index contrast between silicon and silicon dioxide (SiO_2_). For the implementation of integrated QKD systems, on-chip devices are needed to accurately manipulate the polarization state of the light injected in photonic-integrated circuits (PICs). Polarizers, polarization beam splitters (PBSs) and polarization rotators (PRs) are employed to this aim. Polarization manipulation in PICs usually relies on an asymmetric waveguide design to allow the optical power transfer between orthogonal polarization modes [14]. Birefringence can be introduced by either acting on the waveguide cross-sectional geometry or on the material stack [15]. 

At low doping, graphene operates as a TE polarizer, its conductivity being dominated by inter-band chiral transitions [16]. When the Fermi level (Ef) is such that Ef > hν/2 (with ν the frequency of the optical wave), graphene behaves similarly to a semi-metal, supporting the TM mode through surface plasmon polariton (SPP) resonances [17]. To set the Fermi energy level of graphene at the value required to switch the polarization, electric field doping can be efficiently employed, as well as the use of ionic liquids for the gating [18], and/or ferroelectric (FE) materials. In these cases, a small external bias induces intense dipole electric fields, suitable to support SPP at telecommunication wavelengths (e.g., 1.55 μm). It is particularly appealing to use FE materials realized by van der Waals heterostructures [19], where the relative orientation between the layers can be engineered to control the electro-optical properties of the heterostructure. High-quality monolayer and multilayer graphene can be prepared by micromechanical cleavage, the so-called scotch tape technique [20,21] or chemical vapor deposition on copper (CVD), which allow one to obtain graphene with high mobility ~1.3 × 10^5^ cm^2^ V^−1^ s^−1^ at a ~10^11^ cm^−2^ electron concentration [22]. In this work, we consider graphene nanoribbons placed on top of a silicon-on-insulator (SOI) strip waveguide with a SiO_2_ buffer layer. A fabrication goal is to ensure a flat SiO_2_ surface for easy graphene transfer and patterning. The graphene transfer is usually efficiently performed using polymer-based pick-up techniques [23]. In previous numerical studies, the graphene layer was either represented by a 2D boundary condition at the interface between neighboring 3D materials, or by a thin 3D slab with complex anisotropic permittivity. Comparable results of the two approaches were found when the thickness of the layer in the volumetric approach did not exceed 1 nm. In addition, perturbation methods provided results which are fully comparable with those obtained by using the 2D boundary condition and the volumetric approach [24]. Recent numerical studies also show how the polarizing behavior of graphene-loaded silicon waveguides is determined by the refractive index of the superstrate cladding material and the waveguide’s height, rather than graphene’s Fermi level [25]. In particular, this study showed that the waveguide under study could not be switched between TE-pass and TM-pass regimes via Fermi-level tuning. It hence evidenced that the waveguide design is essential for the development of optimized TE- or TM-pass polarizers, since it allows to control the fraction of the electric field which aligns tangentially to the graphene layer.

In this work, we now propose a polarization control technique using graphene nanoribbons. In graphene–silicon heterostructures, optical absorption is allowed only for the fraction of the electric field that is parallel to the graphene plane, and the birefringent behavior thus takes place because this fraction is generally different for the TE and TM modes in silicon nanophotonic waveguides [23,24,25]. Great efforts are currently underway to prepare, at an industrial scale, single- and bilayer graphene, by means of chemical exfoliation [26,27] and chemical vapor deposition [23], for their subsequent implementation in silicon–graphene hybrid integrated photonic devices for quantum photonics applications. In this context, we are exploring the possibility of controlling the state of polarization of light beams in silicon waveguides with graphene nanoribbons. Graphene can selectively support polarization modes either depending on its Fermi level, on the geometry layout of the waveguide or on the photon energy, with the additional advantage of broadband operation because of its gapless optical spectrum [28]; recent work shows also that the chiral nature of 2D materials, such as graphene and van der Waals heterostructures, can be used to control photon polarization, in a wide frequency range, envisioning also promising developments in gate-controlled polarizer [29]. 

## 2. Device Layout and Design Strategy

The most common platform for PIC is silicon-on-insulator (SOI), constituted by a thin layer of Si lying on a thick (typically > 1 μm) layer of SiO_2_ deposited on a bulk Si wafer. All the standard optical components (grating couplers, waveguides, splitters, filters, interferometers and resonators) are usually realized on the same PIC on a standard 220-nm-thick SOI platform. A critical task to be accomplished is the coupling of the light from (to) an optical fiber to (from) the PIC. 

**Figure 1:**
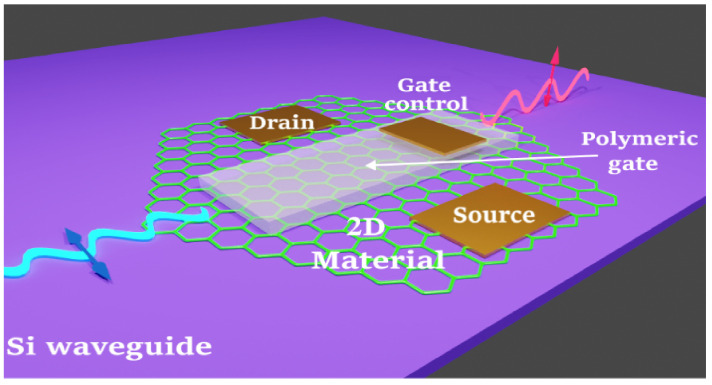
Pictorial view of a nanodevice architecture that may act as the building block for the envisioned 2D material-based polarization processor. The two-dimensional material (graphene) lies on the Si waveguide and is coupled to it. In addition, it can be electrically contacted by two ohmic contacts (source–drain) and equipped with a soft-matter component enabling the ionic gate control of the chemical potential in the nanomaterial.

Figure 1 provides a schematic view of the architecture of a complete graphene-based polarization processor, which exploits a graphene sheet coupled to a Si waveguide. The drain and source electrodes operate the electro-optical modulation, and the gate electrode controls and tunes the 2D electron charge density in graphene.

In this work, we numerically analyze graphene’s capability of controlling the TE–TM polarization of light beams traveling down a coupled graphene-silicon waveguide. For this purpose, 2D simulations of the fundamental modes propagating in the waveguide under study have been carried out to characterize the different coupling of the modal electric field components in parallel and orthogonal directions with respect to the graphene sheet.

**Figure 2:**
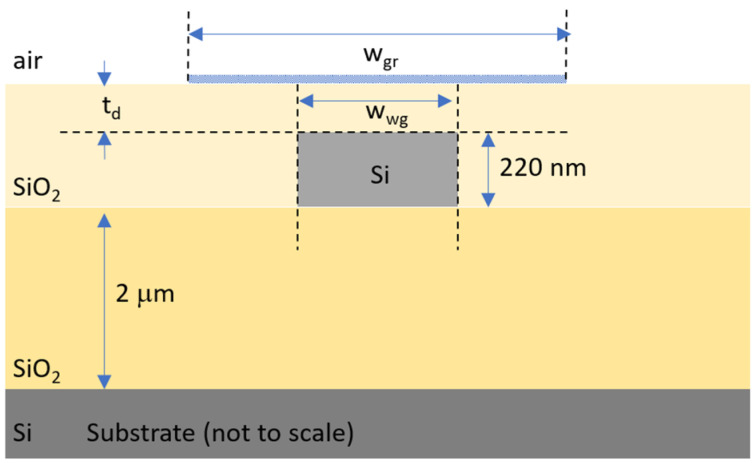
Cross-section of the graphene-on-silicon waveguide used in the 2D simulations to study polarization control.

In Figure 2, we show a typical cross-section of the device under study, with the three investigated geometrical parameters (w_wg_, w_gr_ and t_d_), i.e., the silicon waveguide width, the graphene nanoribbon width and the oxide spacer thickness between silicon and graphene. A standard silicon-on-insulator (SOI) photonic waveguide is optically coupled to a graphene nanoribbon placed on top of the silicon layer. The upper oxide cladding provides electrical insulation between the graphene and silicon layers, with the possibility to locally create flat surfaces for easier transfer of graphene nanoribbons [30]. In this way, the proposed waveguide design potentially lends itself to different graphene fabrication alternatives, ranging from the prototyping-oriented tape-assisted transfer to more scalable chemical vapor deposition (CVD) techniques. In our numerical study, we used a silicon waveguide with a standard width w_wg_ of 450 nm and narrow 300 nm value, changing the graphene width w_gr_ from 50 nm up to 3 μm and the top oxide thickness t_d_ from 10 nm up to 700 nm. 

We performed optical device-level electromagnetic simulations with the Lumerical MODE Optical Waveguide and Coupler Solver [31]. In this numerical analysis method, graphene is modeled as a 2D boundary condition characterized by surface conductivity described with the Kubo formalism [24,25]:

$$\sigma_{s}\left(\omega ,E_{F},\tau ,\;T\right)\approx \frac{e^{2}E_{F}}{\pi \hbar^{2}}\frac{i}{i/\tau +\omega }$$

$$+\frac{e^{2}}{4\hbar }\left\{\frac{1}{2}\left[\tanh\left(\frac{\hbar \omega +2E_{F}}{4k_{B}T}\right)+\tanh\left(\frac{\hbar \omega -2E_{F}}{4k_{B}T}\right)\right]-\frac{i}{2\pi }\ln\left[\frac{{\left(\hbar \omega +2E_{F}\right)}^{2}}{{\left(\hbar \omega +2E_{F}\right)}^{2}-{\left(2k_{B}T\right)}^{2}}\right]\right\}$$

where ω, $E_{F}$, τ, T, $k_{B}$ and ℏ are the circular frequency of light, the energy of the Fermi level in the graphene layer, the time constant corresponding to the graphene electronic relaxation time, the absolute temperature, the Boltzmann constant and the reduced Planck constant, respectively. We used the following values in our simulations: ω = 1.216 × 10^15^ rad·s^−1^ (corresponding to the wavelength λ = 1.55 μm), τ = 0.2 ps and T = 300 K. The graphene Fermi level was varied between 0 and 1 eV, i.e., in the range that can be experimentally modified by applying a voltage and/or doping. The refractive indices of silicon and silica at the wavelength of 1.55 μm were taken as 3.47 and 1.44, respectively. The simulations were performed using a coarse mesh (maximum step: 10 nm) for the calculations in the substrate and waveguide region and a much finer mesh (maximum step: 0.25 nm) for the graphene sheet.

## 3. Results and Discussion

**Figure 3:**
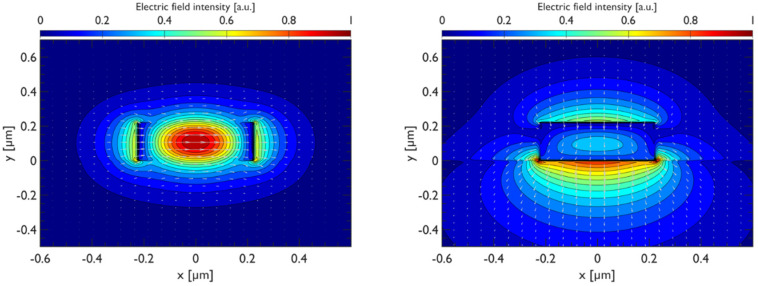
(**Left**): The optical modes supported by a standard 450 nm × 220 nm SOI waveguide. (**Left**): Transverse electric (TE) mode. (**Right**): Transverse magnetic (TM) mode.

We began the investigation by simulating the profile of the TE and TM modes in an SOI waveguide with dimensions 450 nm × 220 nm, which is the typical size used for applications in silicon quantum photonics. Silicon-on-insulator (SOI) waveguides suffer from large polarization birefringence, because of the high refractive index contrast between silicon and silicon dioxide SiO_2_. We can see in Figure 3 that, without graphene, the TE mode is almost symmetrically confined in the *y* direction, while the TM mode in the same direction spreads further. 

**Figure 4:**
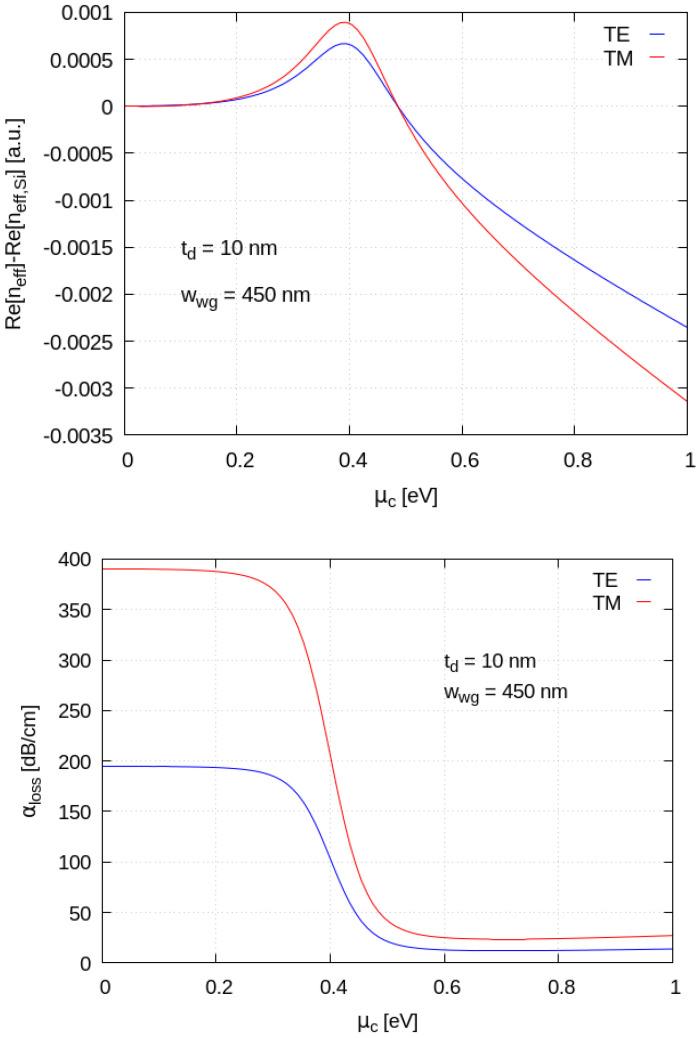
(**Top**): Normalized effective index of the TE and TM modes versus graphene chemical potential (*µ_c_*). (**Bottom**): Optical absorption of the TE and TM modes varying versus *µ_c_*. Light wavelength was set to 1.55 μm, graphene electronic relaxation time *τ* = 20 fs.

The presence of the graphene sheet modifies the propagation properties of these two modes, because of their overlap with the 2D material. The mode properties have been found to be strongly dependent on Graphene’s chemical potential. Figure 4 shows the relative difference of modal effective refractive indices (top panel) and optical absorption per-unit-length losses (bottom panel) as a function of Graphene’s chemical potential normalized to the case of the same silicon waveguide without nanoribbon on top. The light wavelength has been set to 1.55 μm to preserve compatibility with mainstream silicon photonic telecom applications which typically harness C-band near-infrared operation. For this simulations, graphene electronic relaxation time was kept fixed to *τ* = 20 fs. We can see that when increasing the chemical potential (*µ_c_*), the normalized effective refractive index of the TM mode grows more rapidly compared to that of the TE one, and that both have a maximum close to 0.4 eV; a further increase in the chemical potential leads to an interesting crossing, with the TE mode index exceeding that of the TM mode. Regarding the absorption losses, we see that at small *µ_c_*, the TE mode suffers much smaller losses compared to the TM one, while, with increasing *µ_c_*, the losses for the two modes progressively reduce and approach one another. The numerical analysis reveals that the *µ_c_* tuning affects the TE and TM polarizations in a similar way, while it has a different effect on the optical loss of the two modes (Figure 4). We found that, in order to reduce the optical losses, reasonable working points for a polarizing device should stay in the range *µ_c_* > 0.5 eV.

**Figure 5:**
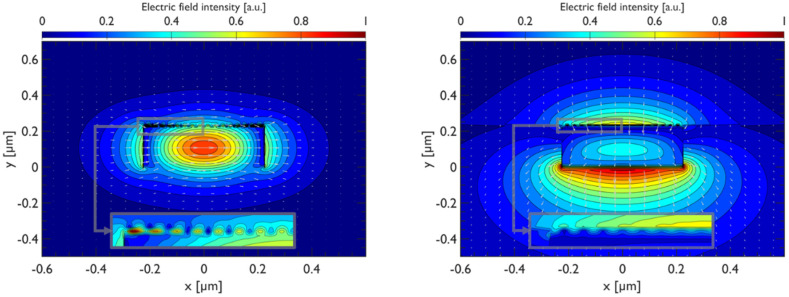
TE (**left panel**) and TM (**right panel**) modes of the SOI waveguides coupled to graphene nanoribbons (*µ_c_* = 1.6 eV).

The spatial distribution of the TE and TM modes at chemical potential *µ_c_* = 1.6 eV is shown in Figure 5. This is an extreme case that has been investigated in the literature [32,33] and which we report for completeness to confirm the onset of surface plasmons in that range of chemical potentials. Nevertheless, in our work, we restrict our study to values of the chemical potential up to 1 eV, since we found it was sufficient to achieve polarization control while keeping the graphene dispersion in the linear regime. 

**Figure 6:**
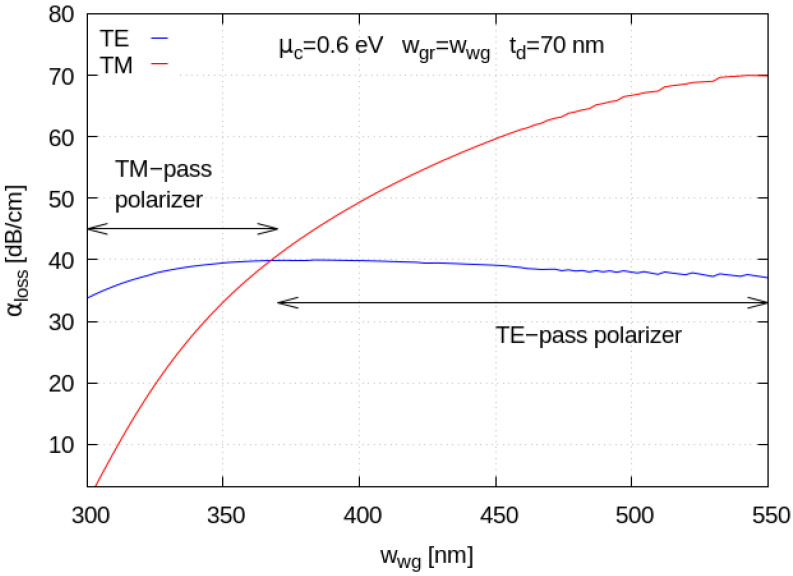
Waveguide loss as a function of the waveguide width. The graphene chemical potential value is taken as *µ_c_* = 0.6 eV and the light wavelength is set to 1.55 μm, with a graphene electronic relaxation time *τ* = 20 fs.

**Figure 7:**
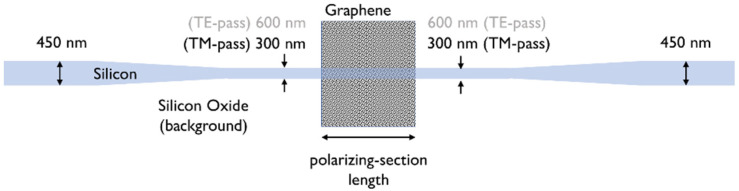
Top view of the graphene-on-silicon waveguide used in the 2D simulations to study polarization control.

In Figure 6, we show the optical absorption loss coefficient for the TE and TM modes as a function of the Si waveguide width. We see that waveguide width tapering enables both TE-pass and TM-pass polarizing functionalities; a possible full layout of a device which can accommodate the TM-pass polarizing functionality by using narrow waveguide widths is sketched in Figure 7. A standard 450 nm-wide waveguide can indeed be narrowed down with conservative linear tapers to make it interact with the deposited Graphene nanoribbon with the modal field distribution required to obtain a TM-pass behavior.

**Figure 8:**
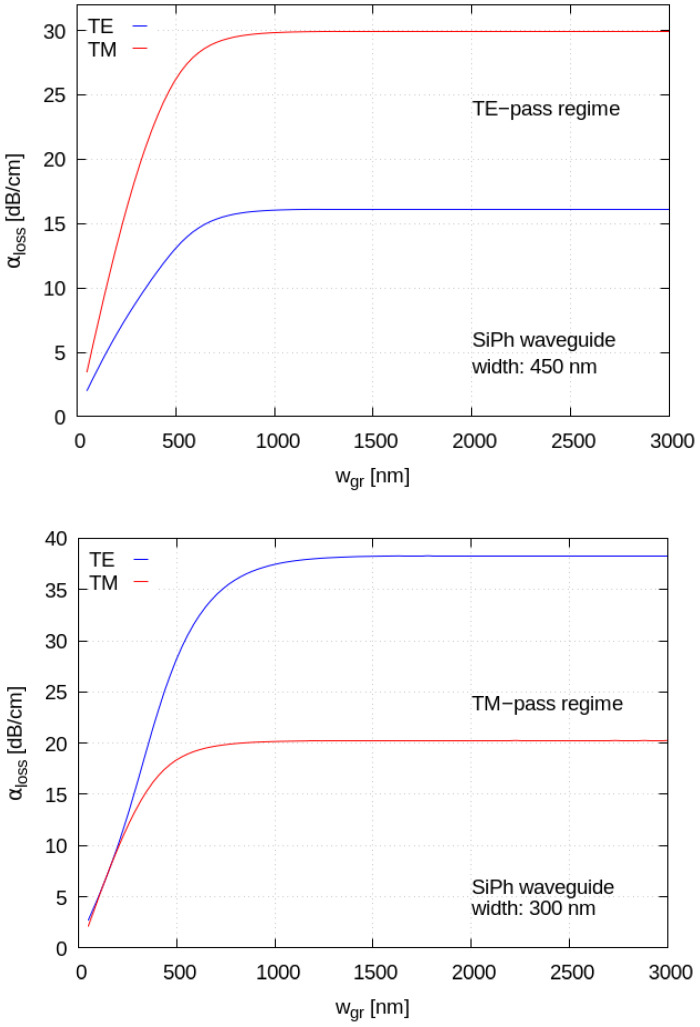
(**Top**): Mode absorption coefficients for a waveguide of width 450 nm. (**Bottom**): Mode absorption coefficients for a waveguide of width 300 nm. The absorption has been calculated as a function of the graphene width.

In Figure 8, we illustrate the system for different widths of the graphene layer, both for the standard and for the narrow waveguide: the overall polarizing behavior is maintained and saturates for graphene widths greater than the transverse optical mode’s dimensions. The polarizer operation results in robust against graphene fabrication widths when working in the “saturated region”, which translates into not too stringent requirements for graphene patterning during lithographic processes.

**Figure 9:**
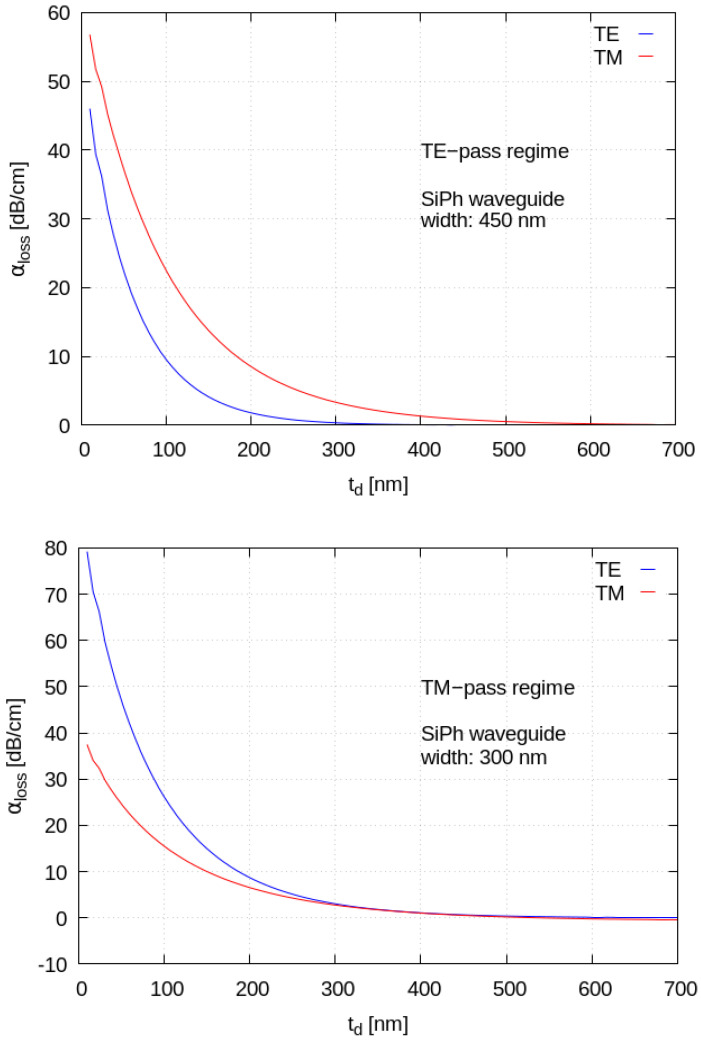
(**Top**): Mode absorption coefficients for a waveguide of width 450 nm. (**Bottom**): Mode absorption coefficients for a waveguide of width 300 nm. The absorption has been calculated as a function of the spacer thickness.

In Figure 9, we show the mode parameters calculated by varying the top oxide thickness from 10 nm to 700 nm, keeping a fixed Si waveguide width and using a graphene width appropriate for working in the “saturation region”. The simulation clarifies how the graphene interaction with the optical modes gradually vanishes when it is moved away from the guided optical modes. If we wish to consider a number of graphene layers greater than one, we should introduce new models for the response of the material. Even in the simplest case of bilayer graphene, the morphological properties change markedly with respect to monolayer graphene [27], and the electronic properties also change, as shown, for instance, in electrical transport experiments in a magnetic field, which allow us to probe the collective response of the electrons and their spin [34]. Therefore, the numerical prediction for a number of layers greater that two requires further modelling of the material and a careful analysis which goes beyond the scope of this work.

In our calculations, we study the behavior of the proposed device by setting the silicon waveguide width, the graphene nanoribbon width and the oxide spacer thickness as design parameters. As already reported in [25], graphene’s physical parameter tuning does not allow us to achieve strong polarization-diverse behavior and a more systematic design approach is thus needed. For this purpose, we set the graphene chemical potential to *µ_c_* = 0.6 eV and the graphene electronic relaxation time to 20 fs, keeping it constant in all simulations, while varying the other geometrical parameters. As a general rule, an increase in graphene chemical potential helps in reducing optical insertion losses, at the expense of weaker interaction strength with the optical mode. Our first results (Figure 6), obtained by fixing the nanoribbon width equal to the waveguide width, show the capability of this device to work as a TM-pass polarizer for a narrow waveguide configuration (300 nm width) and as a TE-pass polarizer for a standard width configuration (450 nm). In addition, keeping the silicon waveguide width and the top oxide thickness fixed, the overall polarizing behavior is maintained and saturates for graphene widths greater than the transverse optical mode’s dimensions (Figure 8).

## 4. Conclusions

In conclusion, we have presented the idea of a new architecture for TE–TM polarization control and tuning in SOI waveguides by means of graphene nanoribbons. The graphene material has been modeled as a two dimensional 2D boundary conditions using the Kubo formula for surface conductivity. By performing numerical analysis, we found that the *µ_c_* tuning similarly affects both TE and TM polarizations, despite inducing different optical losses. We demonstrate that to reduce the optical losses, reasonable working points are the ones with the graphene chemical potential *µ_c_* > 0.5 eV. Another interesting and applicable result is that, when increasing the thickness of the oxide spacer between the silicon waveguide and of the top graphene layer, the device insertion losses are reduced, maintaining a reasonable polarization extinction ratio. The tapering of a standard waveguide can then be harnessed to create devices with TE-pass or TM-pass polarizing functionalities. These results are applicable to silicon photonics polarization-encoded QKD devices working in the telecom 1.55 μm C-band.
